# Doubly robust estimator of risk in the presence of censoring dependent on time-varying covariates: application to a primary prevention trial for coronary events with pravastatin

**DOI:** 10.1186/s12874-020-01087-8

**Published:** 2020-07-31

**Authors:** Takuya Kawahara, Tomohiro Shinozaki, Yutaka Matsuyama

**Affiliations:** 1 Clinical Research Promotion Center, The University of Tokyo Hospital, 7-3-1, Hongo, Bunkyo-ku, Tokyo, 113-8655 Japan; 2 Department of Information and Computer Technology, Graduate School of Engineering, Tokyo University of Science, Tokyo, Japan; 3 Department of Biostatistics, School of Public Health, The University of Tokyo, Tokyo, Japan

**Keywords:** Double robustness, Dependent censoring, Prediction, Time-varying covariate

## Abstract

**Background:**

In the presence of dependent censoring even after stratification of baseline covariates, the Kaplan–Meier estimator provides an inconsistent estimate of risk. To account for dependent censoring, time-varying covariates can be used along with two statistical methods: the inverse probability of censoring weighted (IPCW) Kaplan–Meier estimator and the parametric g-formula estimator. The consistency of the IPCW Kaplan–Meier estimator depends on the correctness of the model specification of censoring hazard, whereas that of the parametric g-formula estimator depends on the correctness of the models for event hazard and time-varying covariates.

**Methods:**

We combined the IPCW Kaplan–Meier estimator and the parametric g-formula estimator into a doubly robust estimator that can adjust for dependent censoring. The estimator is theoretically more robust to model misspecification than the IPCW Kaplan–Meier estimator and the parametric g-formula estimator. We conducted simulation studies with a time-varying covariate that affected both time-to-event and censoring under correct and incorrect models for censoring, event, and time-varying covariates. We applied our proposed estimator to a large clinical trial data with censoring before the end of follow-up.

**Results:**

Simulation studies demonstrated that our proposed estimator is doubly robust, namely it is consistent if either the model for the IPCW Kaplan–Meier estimator or the models for the parametric g-formula estimator, but not necessarily both, is correctly specified. Simulation studies and data application demonstrated that our estimator can be more efficient than the IPCW Kaplan–Meier estimator.

**Conclusions:**

The proposed estimator is useful for estimation of risk if censoring is affected by time-varying risk factors.

## Background

Establishment of the long-term effectiveness of primary prevention treatments often requires large randomized controlled trials (RCTs) over a long time period. In such RCTs, survival functions and risks between randomized groups are compared using the Kaplan–Meier estimator because censoring before the end of the follow-up cannot be avoided. This approach assumes independent censoring, such that censoring occurs randomly in each treatment group. The standardization approach can provide a consistent estimate of risks in each group even if censoring is not unconditionally independent, but the conditionally independence of potential survival time after stratification of treatment groups and baseline covariates [1–4]. In this paper, we call this type of censoring as baseline-conditional independent censoring.

Even a baseline-conditional independent censoring assumption can be dubious. Our motivating study is the Management of Elevated Cholesterol in the Primary Prevention Group of Adult Japanese (MEGA) study, which is a large primary prevention RCT for coronary heart disease (CHD) using pravastatin, where censoring before the end of follow-up occurred in about 10% of patients [5]. Patients enrolled in the MEGA study had hypercholesterolemia (total cholesterol (TC) level: 220–270 mg/dl), were 40–70 years old, and received daily clinical care during the follow-up period. When a patient with hypercholesterolemia received a medical checkup and found that their plasma lipids were worsening (e.g., increasing TC), they may have required other drugs that were not allowed in the study protocol. Patients who observed worsening of their symptoms might go to see a doctor other than their primary care doctor. These cases may have led to censoring dependent on mid-course clinical characteristics, and the censoring was correlated with future CHD events. If censoring is dependent on potential survival time even after stratification of treatment groups and baseline covariates, the Kaplan–Meier estimator provides inconsistent estimates of survival function [6]. In such a situation, one possibility to mitigate the dependency is to use time-varying covariates measured during the follow-up period.

The inverse probability of censoring weighted (IPCW) Kaplan–Meier estimator is a semiparametric method for estimation of risk that adjusts for censoring that may depend on the observed past [7]. It requires fitting a model for the probability of censoring at each time conditional on past covariates. Calculation of the IPCW Kaplan–Meier estimate needs to update censoring probability at each time and to weight each subject in the risk set. The weight depends on the time-varying covariates, but not on the future prognosis. The drawback of the IPCW estimator is that it can be statistically inefficient [8].

An alternative to IPCW methods is a g-formula-based estimator, which can be estimated using two different principles. First representation of the g-formula is an iterated conditional expectation, and targeted maximum likelihood estimation can be applied, which was first introduced by Bang and Robins [9]. Their method uses the weight of the IPCW method and regression models for the outcome process. It can produce doubly robust estimates, meaning that the estimator is consistent if either the regression model for the hazard of censoring or a regression model for the outcome process is correctly specified, but necessarily both [10–12]. However, only a few researchers have applied this method. One of the reasons may be that they are unintuitive because it requires recursive regression models for an iterated conditional expectation; first, we regressed the outcomes measured at *t* = *K* on the covariates measured up to *t* = *K* - 1, second, we regressed the predicted outcome on the covariates measured up to *t* = *K* - 2, and we continue these procedures until *t* = 1. The second representation of the g-formula is the generalized version of standardization [1, 2], and the parametric g-formula estimator (g-computation algorithm formula) can be applied. The parametric g-formula estimator requires models for the outcome and covariate process [13]. It can be regarded as a sequential, non-recursive imputation-based methodology [14, 15], so it is intuitive for applied researchers. It is flexible because it can easily compare dynamic treatment regimens [16]. However, it requires a specification of full-model likelihood, and robustness regarding model correctness can be a concern. A doubly robust estimator for the parametric g-formula estimator, involving the time-varying covariates, has not been proposed.

In this paper, we propose an extension of the parametric g-formula estimator that is more robust at modeling misspecification. The key idea is to combine the IPCW estimator and the parametric g-formula estimator into doubly robust estimators [9, 17–19] while incorporating time-varying covariates to adjust for dependent censoring.

The paper is organized as follows. In the next section, we briefly describe the MEGA study and introduce notations and assumptions. We also describe our proposed estimator, and we give settings and the results of simulation studies. Finally, the proposed estimator is applied to the MEGA study data.

## Methods

### Data, notations, and assumptions

The MEGA study is a prospective, randomized, open-label, blinded-endpoint-designed controlled trial conducted in Japan to evaluate the primary preventive effect of pravastatin against CHD in daily clinical practice. A total of 7832 men and postmenopausal women aged 40–70 years with hypercholesterolemia and no history of CHD or stroke were randomized to dietary therapy only (diet group) or dietary therapy plus 10–20 mg daily pravastatin (diet plus pravastatin group) between February 1994 and March 1999.

After randomization, laboratory tests were conducted at months 1, 3, and 6, and annually thereafter. The follow-up period was initially scheduled for 5 years. Table 1 shows the types and number of events within 5 years. Although there were three reasons for censoring during the study period (refusal of follow-up, death by causes other than CHD, and loss to follow-up), we collectively treated them as censoring before the end of the follow-up period.

**Table 1:** Type and number of events within 5 years in the MEGA study

	Diet group		Diet + pravastatin group	
	*n*	%	*n*	%
CHD event	85	2.1	57	1.5
Follow-up completed	3498	88.2	3353	86.7
Refusal of follow-up	259	6.5	364	9.4
Death by causes other than CHD	60	1.5	42	1.1
Loss to follow-up	64	1.6	50	1.3
Total	3966	100.0	3866	100.0

Let *t* = 1, …, *T* denote month of follow-up where *T* + 1 = 60 months is the follow-up of interest. There were 7832 patients at baseline, and observations of patients were assumed to be independently identically distributed. *R* denotes the treatment assigned (*R* = 1 for assignment to the diet plus pravastatin group, and *R* = 0 for assignment to the diet group). *C*_*t*_ and *Y*_*t*_ denote the indicator of censoring and occurrence of a CHD event by time *t,* respectively, with *C*_0_ = *Y*_0_ = 0 by definition. *L*_*t*_ denotes time-varying covariates measured at time *t*, and *V* denotes baseline covariates that are time-independent (e.g. sex, current smoker). We assumed that baseline covariates *V* and *L*_0_ are always observed. We denoted the history of a variable using overbars. For example, ${\overline{L}}_t=\left({L}_0,\dots, {L}_t\right)$ is the covariate history through time *t*. We assumed the order (*C*_*t*_, *Y*_*t*_, *L*_*t*_) within each interval (*t* - 1, *t*); therefore, *Y*_*t*_ and following variables are missing if *C*_*t*_ = 1. We defined *C*_*T* + 1_ = 1 if *C*_*T*_ = *Y*_*T*_ = 0 (follow-up completed).

We wanted to estimate the marginal event-free survival in each treatment group if any censoring was absent in the study population. However, observed data contains censoring, as in the MEGA study. The usual the Kaplan–Meier estimator assumes independent censoring, that is, the hazard of *Y*_*t*_ among subjects at risk is the marginal hazard of *Y*_*t*_ given the treatment group. The standardization approach, or a g-formula that adjusts for baseline covariates, assumes baseline-conditional independent censoring, that is, the hazard of *Y*_*t*_ among subjects at risk is the conditional hazard of *Y*_*t*_ given the treatment group and baseline covariates [1–4].

Even when these two assumptions are attainable, estimators discussed in the next section provide a consistent estimate of the marginal survival in each treatment group if any censoring was absent in the study population. These estimators assume positivity (Eq. 1) and are conditionally independent of censoring (Eq. 2);

1 $$ \Pr \left({C}_t=0\left|{\overline{C}}_{t-1}=0,{\overline{Y}}_{t-1}=0,R,V,\right.\ {\overline{L}}_{t-1}\right)>0\;\mathrm{for}\ t=1,2,\dots, T $$

2 $$ {\displaystyle \begin{array}{c}\Pr \left({C}_t=0\left|{\overline{C}}_{t-1}=0,{\overline{Y}}_T=0,R,V,{\overline{L}}_{t-1}\right.\right)=\Pr \left({C}_t=0\left|{\overline{C}}_{t-1}=0,{\overline{Y}}_{t-1}=0,R,V,{\overline{L}}_{t-1}\right.\right)\\ {}\mathrm{for}\ t=1,2,\dots, T\end{array}}. $$

The conditional independence of censoring assumption, Eq. (2), states that for *t* = 1, 2, … *T*, the variables (*Y*_*t*_, …, *Y*_*T*_) is independent of *C*_*t*_, in other words, the distribution of (*Y*_*t*_, …, *Y*_*T*_) is the same between *C*_*t*_ = 1 and *C*_*t*_ = 0 among subjects who had a similar history of the covariates. The conditionally independence of censoring assumption is also referred to as no unmeasured confounders for the censoring assumption [7], which states that conditional on the treatment groups, baseline covariates, and the time-varying covariates measured until time *t* - 1, the hazard of censoring at time *t* does not further depend on unmeasured confounders for censoring and unobserved CHD. In the next section, we describe the existing estimators and our proposed estimator for the hazard of *Y*_*t*_.

### Existing estimators and proposed estimator

Due to randomization, baseline factors are balanced between treatment groups. In this section, we focus on the diet plus pravastatin group (*R* = 1) and suppress *R* for notational simplicity. A similar argument holds for the diet group (*R* = 0).

#### Estimators of hazard at t = 1

At time *t* = 1, the observed data is *n* copies of (*V*, *L*_0_, *C*_1_, (1 - *C*_1_)*Y*_1_). We show three types of estimators for Pr(*Y*_1_ = 1); the IPCW estimator, the parametric g-formula estimator, and the doubly robust estimator.

To obtain the IPCW estimate, we need to fit a model for *C*_1_ such as the logistic model Pr(*C*_1_ = 0|*V*, *L*_0_; *α*) = *e*(*V*, *L*_0_; *α*) = {1 + exp(−*α*_0_ – *α*_1_*V* – *α*_2_*L*_0_)}^− 1^. After fitting the model, the IPCW estimator for Pr(*Y*_1_ = 1) is expressed as ${n}^{-1}\sum \limits_i\left(1-{C}_{i1}\right){Y}_{i1}/e\left({V}_i,{L}_{i0};\hat{\alpha}\right)$. The consistency of the IPCW estimator relies on the correct specification of *e*(*V*, *L*_0_; *α*). If no censoring is observed at *t* = 1, we set $e\left({V}_i,{L}_{i0};\hat{\alpha}\right)=1$; therefore, the IPCW estimator equals the empirical risk.

To obtain the parametric g-formula estimate, we need to fit a model for *Y*_1_ such as the logistic model Pr(*Y*_1_ = 1|*C*_1_ = 0, *V*, *L*_0_; *β*) = *p*(*V*, *L*_0_; *β*) = {1 + exp(−*β*_0_ – *β*_1_*V* – *β*_2_*L*_0_)}^− 1^. After fitting the model for the subjects not censored at *t* = 1 (subjects with *C*_1_ = 0), the parametric g-formula estimator for Pr(*Y*_1_ = 1) is expressed as ${n}^{-1}\sum \limits_ip\left({V}_i,{L}_{i0};\hat{\beta}\right)$. The consistency of the parametric g-formula estimator relies on the correct specification of *p*(*V*, *L*_0_; *β*).

To obtain the doubly robust estimate, we need to fit a model for *C*_1_ and *Y*_1_ similarly as conducted for the IPCW estimator and the parametric g-formula estimator, respectively. After fitting the models *e*(*V*, *L*_0_; *α*) and *p*(*V*, *L*_0_; *β*), the doubly robust estimator for Pr(*Y*_1_ = 1) is expressed as

3 $$ {n}^{-1}{\Sigma}_i\left[\frac{\left(1-{C}_{i1}\right){Y}_{i1}}{e\left({V}_i,{L}_{i0};\hat{\alpha}\right)}-\frac{\left(1-{C}_{i1}\right)-e\left({V}_i,{L}_{i0};\hat{a}\right)}{e\left({V}_i,{L}_{i0};\hat{\alpha}\right)}p\left({V}_i,{L}_{i0};\hat{\beta}\right)\right]. $$

The contributions of censored patients or patients with an event are different; for censored patients, their contribution is $p\left({V}_i,{L}_{i0};\hat{\beta}\right)$ like the g-formula estimator, and for patients with an event, their contribution is ${Y}_{i1}/e\left({V}_i,{L}_{i0};\hat{\alpha}\right)-\left\{1-e\left({V}_i,{L}_{i0};\hat{\alpha}\right)\right\}p\left({V}_i,{L}_{i0};\hat{\beta}\right)/e\left({V}_i,{L}_{i0};\hat{\alpha}\right)$. The doubly robust estimator is consistent if either the model *e*(*V*, *L*_0_; *α*) or *p*(*V*, *L*_0_; *β*) is correctly specified [9, 17–19]. Intuitively, when the model for censoring is correctly specified, the term $\left(1-{C}_{i1}\right)-e\left({V}_i,{L}_{i1};\hat{\alpha}\right)$ should be zero, so (3) reduces to the IPCW estimator and is, therefore, consistent. Inside the summation can be expressed as $\left(1-{C}_{i1}\right)\left\{{Y}_{i1}-p\left({V}_i,{L}_{i0};\hat{\beta}\right)\right\}/e\left({V}_i,{L}_{i0};\hat{\alpha}\right)+p\left({V}_i,{L}_{i0};\hat{\beta}\right)$, and when the model for an event is correctly specified, the term ${Y}_{i1}-p\left({V}_i,{L}_{i0};\hat{\beta}\right)$ should be zero, so (3) reduces to the g-formula estimator and is, therefore, consistent. Our proposed estimator utilizes this doubly robust estimator for the hazard of *Y*_1_. In the next subsection, we show how to extend it to estimate the hazard of *Y*_*t*_ (*t* > 1) incorporating time-varying covariates.

We noted that with one categorical baseline covariate and no parametric model needed for outcomes and censoring, it can be shown that the IPCW estimator, the g-formula estimator, and the doubly robust estimator are equivalent. Specifically, given *n* subjects, all of whom may be stratified into *j* levels of a baseline covariate, such that *a*_*j*_, *m*_*j*_, and *n*_*j*_ are the number observed (i.e. not censored), number of events, and overall number at level *j* of the covariate, respectively. The IPCW estimator can be written as (1/*n*)*Σ*_*j*_*m*_*j*_*/ (a*_*j*_*/ n*_*j*_*)* = (1/*n*) *Σ*_*j*_*n*_*j*_*m*_*j*_*/ a*_*j*_, because Pr(*C*_1_ = 0| level *j*) = *a*_*j*_*/ n*_*j*_. The g-formula estimator can be written as (1/*n*) *Σ*_*j*_*n*_*j*_ (*m*_*j*_*/ a*_*j*_*)*, because Pr(*Y*_1_ = 1| level *j*) = *m*_*j*_*/ a*_*j*_. Finally, the doubly robust estimator can be written as (1/*n*) *Σ*_*j*_ [*m*_*j*_*/ (a*_*j*_*/ n*_*j*_*)* – {(*n*_*j*_ - *a*_*j*_) (0 - *a*_*j*_*/ n*_*j*_) / (*a*_*j*_*/ n*_*j*_) + *a*_*j*_ (1 - *a*_*j*_*/ n*_*j*_) / (*a*_*j*_*/ n*_*j*_)} (*m*_*j*_*/ a*_*j*_)] = (1/*n*) *Σ*_*j*_*n*_*j*_*m*_*j*_*/ a*_*j*_, which is exactly a common form as the IPCW estimator and the g-formula estimator.

#### Estimators of hazard at t > 1

In this subsection, we show the estimators of the hazard of *Y*_*t*_ (*t* > 1), which are extended versions of the IPCW estimator and the parametric g-formula estimators for Pr(*Y*_1_ = 1). Finally, we propose a doubly robust estimator that extends Eq. (3).

To obtain the IPCW Kaplan–Meier estimate, we need to fit a model for *C*_*t*_ such as the pooled logistic model,

4 $$ \mathrm{logit}\;\Pr \left({C}_t=0\left|{\overline{C}}_{t-1}=0,{\overline{Y}}_{t-1}=0,V,{\overline{L}}_{t-1}\right.\right)={\alpha}_{0t}+{\alpha}_1V+{\alpha}_2{L}_{t-1}. $$

In the model, it is possible to include *L*_0_*, …, L*_*t* – 2_, but in some cases, it may cause multicollinearity due to the correlation between *L*_0_*, …, L*_*t* - 1_. After fitting the model using the maximum likelihood estimation, the IPCW Kaplan–Meier estimator for the hazard of *Y*_*t*_ is expressed as $\hat{\Pr}\left({Y}_t=1|{\overline{Y}}_{t-1}=0\right)={\sum}_i{Y}_{it}{\pi}_{it}\left(\hat{\alpha}\right)/{\sum}_i{X}_{it}{\pi}_{it}\left(\hat{\alpha}\right)$, where ${\pi}_t\left(\hat{\alpha}\right)$ is obtained as

$$ {\pi}_t\left(\hat{\alpha}\right)=\prod \limits_{j=1}^t\Pr \left({C}_j=0|{\overline{C}}_{j-1}=0,{\overline{Y}}_{j-1}=0,V,{\overline{L}}_{j-1};\hat{\alpha}\right), $$

and *X*_*t*_ is the at-risk indicator, which is 1 if the patient is at-risk at time *t* and is 0 otherwise. Finally, the risk at *t* can be obtained as $1-\prod \limits_{j=1}^t\left\{1-\hat{\Pr}\left({Y}_j=1|{\overline{Y}}_{j-1}=0\right)\right\}$. The consistency of the IPCW Kaplan–Meier estimator relies on the correct specification of the model for *C*_*t*_ (Eq. 4) [7]. Note that the IPCW Kaplan–Meier estimator reduces to the usual Kaplan–Meier estimator when *α*_1_ and *α*_2_ of Eq. (4) are 0, that is, the independent censoring assumption is true [20].

To obtain the parametric g-formula estimate, we need to fit a model for *Y*_*t*_. Unlike baseline covariates, time-varying covariates will not be measured for patients who were censored before time *t*. Thus, we need to specify the full-model likelihood (likelihood for conditional event probability and time-varying covariates) by fitting models for *Y*_*t*_ and *L*_*t*_ such as

5 $$ \mathrm{logit}\;\Pr \left({Y}_t=1|{\overline{C}}_t=0,{\overline{Y}}_{t-1}=0,V,{\overline{L}}_{t-1}\right)={\beta}_{0t}+{\beta}_1V+{\beta}_2{L}_{t-1}, $$

and

6 $$ E\left({L}_t|{\overline{C}}_t=0,{\overline{Y}}_{t-1}=0,V,{\overline{L}}_{t-1}\right)={\gamma}_{0t}+{\gamma}_1V+{\gamma}_2{L}_{t-1}. $$

After fitting the models using the maximum likelihood estimation, we sequentially imputed the conditional probability of CHD event and time-varying covariates from *t* = 1 to *T*. The parametric g-formula estimator for the risk at *t* can be obtained as ${n}^{-1}{\sum}_{j=1}^t{\sum}_i{m}_{\mathrm{ij}}\left(\hat{\beta},\hat{\gamma}\right)$, where ${m}_t\left(\hat{\beta},\hat{\gamma}\right)$ is obtained as ${m}_t\left(\hat{\beta},\hat{\gamma}\right)=\Pr \left({Y}_t=1|{\overline{Y}}_{t-1}=0,V,{\overline{L}}_{t-1};\hat{\beta},\hat{\gamma}\right){\prod}_{j=1}^{t-1}\left\{1-\Pr \left({Y}_j=1|{\overline{Y}}_{j-1}=0,V,{\overline{L}}_{j-1};\hat{\beta},\hat{\gamma}\right)\right\}$. The consistency of the parametric g-formula estimator relies on the correct specification of the model for *Y*_*t*_ (Eq. 5) and the model for *L*_*t*_ (Eq. 6) [16, 21, 22].

We propose an estimator of the hazard of *Y*_*t*_ that extends the doubly robust estimator (Eq. 3). To obtain the estimate, we need to fit models for *C*_*t*_, *Y*_*t*_, and *L*_*t*_ as conducted for the IPCW Kaplan–Meier estimator (Eq. 4) and the parametric g-formula estimator (Eqs. 5 and 6). After fitting these models, the proposed doubly robust cf *Y*_*t*_ is expressed as,

7 $$ \hat{\Pr}\left({Y}_t=1|{\overline{Y}}_{t-1}=0\right)={\left({\sum}_i{Z}_{it}\right)}^{-1}{\sum}_i\left[\frac{\left(1-{C}_{it}\right){Y}_{it}{Z}_{it}}{\pi_{it}\left(\hat{\alpha}\right)}-\frac{\left(1-{C}_{it}\right)-{\pi}_{it}\left(\hat{\alpha}\right)}{\pi_{it}\left(\hat{\alpha}\right)}\Pr \left({Y}_{i,t}=1|{\overline{Y}}_{i,t-1}=0,{V}_i,{\overline{L}}_{i,t-1};\hat{\beta},\hat{\gamma}\right){Z}_{it}\right] $$

where *Z*_*t*_ is the at-risk or censored indicator, which is 1 if the patient is at-risk at time *t* or censored by *t* and is 0 otherwise. The contributions of patients censored by *t* or patients with an event at *t* are different; for censored patients, their contribution is $\Pr \left({Y}_t=1|{\overline{Y}}_{t-1}=0,V,{\overline{L}}_{t-1};\hat{\beta},\hat{\gamma}\right)$. For patients with an event, their contribution of an event is weighted by the inverse probability of uncensored until *t*. Finally, the risk at *t* is obtained as $1-{\prod}_{j=1}^t\left\{1-\hat{\Pr}\left({Y}_j=1|{\overline{Y}}_{j-1}=0\right)\right\}$. The weights and predicted event probabilities are similar to the ones used in the IPCW Kaplan–Meier estimator and the parametric g-formula estimator, but we need to calculate the function (7) and risk at *t*. As demonstrated in the Additional file 1 (Appendix A), this estimator is consistent if either the model for *C*_*t*_ (Eq. 4) or models for *Y*_*t*_ and *L*_*t*_ (Eq. 5 and 6) is correctly specified. In the IPCW Kaplan–Meier estimator, patients with *C*_*t*_ = 1 were out of the risk set; therefore, they do not contribute to the estimation of the hazard of *Y*_*t*_. On the other hand, patients with *C*_*t*_ = 1 contribute to the estimation of the hazard of *Y*_*t*_ in Eq. (7), which might lead to statistical efficiency. The variance estimate of the proposed estimator can be obtained through a nonparametric bootstrap [23]. We have provided a SAS code for the proposed estimator in an Additional file 1 (Appendix B).

#### Comparison with existing doubly robust estimators

In this subsection, we briefly compare our proposed estimator (7) with existing doubly robust estimators [9, 24, 25]. Zhang et al. [24] and Bai et al. [25] proposed doubly robust estimators for survival functions, which can be summarized as follows:

- *Confounding between treatment groups*: present due to the observational study setting
- *Censoring mechanism*: baseline-conditional independent censoring (censoring may depend only on the baseline covariates)

On the other hand, we proposed an estimator for survival functions,

- *Confounding between treatment groups*: absent due to randomization
- *Censoring mechanism*: conditional independent censoring (censoring may depend on time-varying covariates)

In RCT settings considered here, where no baseline confounding occurs between the treatment groups, the proposed estimator that specifies an empty set as *L*_*t*_ (thus models are unnecessary for the joint density of *L*_*t*_) essentially results in the existing doubly robust estimators provided in [24, 25]. In other words, these existing estimators assume a baseline-conditional independent censoring mechanism, although they also attempt to adjust for baseline-confounding between the groups in observational-study settings.

Bang et al. [9] proposed a doubly robust estimator for the g-formula represented by an iterated conditional expectation. The estimator needs recursive fitting of the iterative conditional expectation. However, as Bang et al. [9] noted, the parametric models can be incompatible with each other, so it is difficult to specify all the models correctly.

### Simulation study

To evaluate the performance of the proposed estimator, we carried out simulation studies with dependent censoring due to a time-varying covariate. We simulated data from two treatment groups, coded as *R* = 0 (control treatment) and *R* = 1 (test treatment). The simulations were based on 1000 replications. We considered the situation where baseline covariates were measured at time *t* = 0, and time-varying covariate and censoring were investigated at time *t* = 1, … 4, on the other hand, event time was measured from time *t* = 0 to *t* = 5 on a continuous time scale. We were interested in the treatment group-specific risks and the risk ratio at *t* = 3 and *t* = 5.

For each patient *i* (= 1, …, 8000), a baseline covariate *V* was generated from the Bernoulli distribution of success probability 0.5. Independently, the time-varying covariate at *t* (= 0, …, 4) was generated from the following mixed effect model,

$$ {L}_{it}=2-0.1\left(1-{R}_i\right)t-0.5{R}_it+{b}_{i0}+{b}_{i1}t+{\epsilon}_{it}. $$

Random variables (*b*_*i*0_, *b*_*i*1_) were generated from a bivariate normal distribution with means of 0 and variance of 1.0 and 0.5, respectively, with a covariance of 0.5. The random error *ϵ*_*it*_ was generated from the standard normal distribution. Distributions of *L*_*t*_ were the same in both treatment groups at *t* = 0 but declined more steeply in the test treatment group such that *L*_*t*_ mimicked TC in the MEGA study.

First, we generated a time to event *T*_*i*_ from the piecewise exponential model, whose hazard function was, for *t* > 0 and *k* ≤ *t* < *k* + 1 (*k* = 0, …, 4),

$$ \lambda \left(t|V,{\overline{L}}_t,R\right)=\exp \left\{-5+1.5V+1.2{U}_k+1.2\left(1-R\right)\right\} $$

where *U*_*t*_ = 1 if *L*_*t*_ < 0, otherwise *U*_*t*_ = 0. Therefore, potential event time was shorter in the control treatment group through the effect of group and time-varying covariate.

Next, we generated censoring *C*_*t*_ at *t* (= 1, …, 4) from the Bernoulli distribution, whose probability was generated using the following logistic model,

$$ \mathrm{logitPr}\left({C}_t=1|{\overline{C}}_{t-1}=0,T>t,V,{\overline{L}}_{t-1},R\right)={\alpha}_0+t+1.5V+1.2{U}_{t-1}+{\alpha}_R\left(1-R\right). $$

${\overline{C}}_4$ = 0 and *T*_*i*_ > 5 indicates that the follow-up was completed. The direct dependence between the event and the censoring time is shown in Additional file 1 (Appendix C).

We considered three scenarios for *α*_0_ and *α*_*R.*_: censoring probabilities in the control and test treatment groups are both 30% (scenario 1), both 20% (scenario 2), and 9 and 12%, respectively (scenario 3). The probabilities in scenario 3 were derived from Table 1.

We created 20,000,000 simulated patients without censoring to calculate the true value of survival probability using their empirical distribution. To understand the performance of estimators, we considered eight situations: all combinations of correct or incorrect censoring models, event models, and covariate models. We defined correct models for censoring, event, and covariate as a model that specified the same covariates with the data-generating model. We defined incorrect models for censoring and event as a model that specified by replacing *U*_*t*_ by exp(*L*_*t*_) without incorporating *V*. An incorrect covariate model was specified without incorporating the interaction term of *b*_*i*1_ and *t.*

Simulations were evaluated in terms of the bias (mean difference between estimated and true parameter value) and relative efficiency (the ratio of the Monte Carlo standard deviation of the IPCW Kaplan–Meier estimator to that of the estimator) of the estimated survival probabilities at time *t* = 3 and *t* = 5.

## Results

### Simulation results

We present our simulation results in Table 2. In Table 2, if the bias exceeded half of the standard error of the estimates, the printed bias was is shown in bold. In scenario 1, the bias for each group at *t* = 5 was seen for the IPCW Kaplan–Meier estimator when the censoring model is incorrect, for the parametric g-formula estimator when one of the event model or covariate model is incorrect. However, our proposed estimator is unbiased when at least one of the censoring model or event model is correctly specified. This result reflected the double robustness of our proposed estimator; when the censoring model or set of event and covariate models are correct, the estimate is unbiased. Unexpectedly, our proposed estimator is less biased than the parametric g-formula estimator, even when the covariate model was incorrect. We consider that this property is only in this simulation because if the covariate model is incorrect, the estimated event probability is also incorrect for true probability. At *t* = 3, the parametric g-formula estimator showed less bias for the test treatment group even when the event model is incorrect. Regarding the bias, similar results can be seen in the other two scenarios.

**Table 2:** Simulation results

Estimator	Model specification			Bias (×100) at *t* = 3			Bias (× 100) at *t* = 5		
	Censoring	Event	Covariate	Control	Test	Log of risk ratio	Control	Test	Log of risk ratio
Scenario 1: 30% censoring in both control and test groups.									
IPCW	Correct	–	–	0.0	0.0	−0.2	− 0.1	0.0	− 0.4
Kaplan–Meier	Incorrect			**0.5**	**0.4**	−0.2	**1.9**	**1.5**	−0.9
Parametric	–	Correct	Correct	0.0 (1.23)	0.0 (1.22)	−0.2 (1.23)	0.0 (1.10)	0.0 (1.08)	−0.3 (1.09)
g-formula		Correct	Incorrect	0.1	0.1	0.1	**1.2**	**0.4**	**3.9**
		Incorrect	Correct	**0.5**	0.1	**3.4**	**2.0**	**1.7**	−2.2
		Incorrect	Incorrect	**0.5**	0.1	**3.4**	**1.9**	**1.7**	−2.4
Proposed	Correct	Correct	Correct	−0.1 (1.04)	0.0 (1.01)	−0.3 (1.02)	− 0.3 (1.00)	− 0.1 (0.99)	− 0.8 (1.01)
doubly robust		Correct	Incorrect	0.0	0.0	− 0.1	0.2	0.0	0.8
		Incorrect	Correct	0.0	0.0	−0.2	0.0	0.0	−0.4
		Incorrect	Incorrect	0.0	0.0	−0.2	0.0	0.0	−0.4
	Incorrect	Correct	Correct	−0.1	0.0	−0.2	− 0.3	−0.1	− 0.7
		Correct	Incorrect	0.0	0.0	−0.1	0.2	0.0	0.9
		Incorrect	Correct	**0.5**	**0.4**	−0.2	**1.9**	**1.5**	−1.0
		Incorrect	Incorrect	**0.5**	**0.4**	−0.2	**1.9**	**1.6**	−0.9
Scenario 2: 20% censoring in both control and test groups.									
IPCW	Correct	–	–	0.0	0.0	−0.3	− 0.1	0.0	− 0.3
Kaplan–Meier	Incorrect			**0.3**	**0.2**	−0.2	**1.3**	**1.0**	−0.5
Parametric	–	Correct	Correct	0.0 (1.25)	0.0 (1.24)	−0.2 (1.26)	0.0 (1.06)	0.0 (1.04)	−0.3 (1.04)
g-formula		Correct	Incorrect	0.0	0.0	0.2	**1.1**	0.3	**3.9**
		Incorrect	Correct	0.1	**−0.2**	**4.2**	**1.4**	**1.2**	−1.4
		Incorrect	Incorrect	0.1	**−0.2**	**4.2**	**1.3**	**1.2**	− 1.6
Proposed	Correct	Correct	Correct	−0.1 (1.03)	0.0 (1.03)	−0.3 (1.05)	− 0.2 (1.00)	− 0.1 (0.99)	−0.6 (1.01)
doubly robust		Correct	Incorrect	0.0	0.0	−0.2	0.1	0.0	0.5
		Incorrect	Correct	0.0	0.0	−0.3	0.0	0.0	−0.3
		Incorrect	Incorrect	0.0	0.0	−0.3	0.0	0.0	−0.3
	Incorrect	Correct	Correct	−0.1	0.0	−0.3	− 0.2	−0.1	− 0.4
		Correct	Incorrect	0.0	0.0	−0.2	0.1	0.0	0.5
		Incorrect	Correct	**0.3**	**0.2**	−0.2	**1.3**	**1.0**	−0.5
		Incorrect	Incorrect	**0.3**	**0.2**	−0.2	**1.3**	**1.0**	−0.5
Scenario 3: 9% censoring in control group and 12% censoring in test group									
IPCW	Correct	–	–	0.0	0.0	−0.3	−0.1	0.0	−0.3
Kaplan–Meier	Incorrect			0.1	0.1	−0.8	**0.5**	**0.6**	−1.6
Parametric	–	Correct	Correct	0.0 (1.26)	0.0 (1.25)	−0.2 (1.28)	0.0 (1.03)	0.0 (1.02)	−0.3 (1.02)
g-formula		Correct	Incorrect	0.0	0.0	0.1	**1.0**	0.2	**3.8**
		Incorrect	Correct	**−0.3**	**−0.5**	3.4	**0.6**	**0.8**	−2.2
		Incorrect	Incorrect	**−0.3**	**− 0.5**	3.3	**0.6**	**0.7**	−2.4
Proposed	Correct	Correct	Correct	0.0 (1.00)	0.0 (1.00)	−0.3 (1.00)	−0.1 (1.00)	− 0.1 (1.00)	−0.4 (1.00)
doubly robust		Correct	Incorrect	0.0	0.0	−0.3	0.0	0.0	0.1
		Incorrect	Correct	0.0	0.0	−0.3	0.0	0.0	−0.3
		Incorrect	Incorrect	0.0	0.0	−0.3	0.0	0.0	−0.3
	Incorrect	Correct	Correct	0.0	0.0	−0.3	−0.1	−0.1	0.0
		Correct	Incorrect	0.0	0.0	−0.3	0.0	0.0	0.1
		Incorrect	Correct	0.1	0.1	−0.8	**0.5**	**0.6**	−1.6
		Incorrect	Incorrect	0.1	0.1	−0.8	**0.5**	**0.6**	−1.6

Numbers in parentheses are the relative efficiency compared with the IPCW Kaplan–Meier estimate with a correctly specified censoring model. If the bias exceeded half of the standard error of the estimates, the printed bias is shown in bold. True values calculated from a large simulated dataset were (0.89, 0.92, 0.69) (at *t* = 3) and (0.81, 0.86, 0.74) (at *t* = 5) for control group, test group, and risk ratio, respectively. The biases (×100) from the method assuming the baseline-conditional independent censoring at *t* = 5 for the control and test groups were (0.5, 0.4) (scenario 1), (0.4, 0.3) (scenario 2), and (0.2, 0.2) (scenario 3)

Regarding the relative efficiency using the IPCW Kaplan–Meier estimator as the reference, both the parametric g-formula estimator and our proposed estimator were more efficient at *t* = 3 than the reference in scenarios 1 and 2. The parametric g-formula estimator was more efficient than the reference even at *t* = 5. However, our proposed estimator had a similar standard error to the reference. In scenario 3, where the censoring probability was the lowest among the scenarios, our proposed estimator had a similar standard error as the reference at both *t* = 3 and 5. The coverage probability of the proposed estimator using the bootstrap method with the correctly specified models was close to the nominal level of 95%. In summary, the efficiency recovery of our proposed estimator may be affected by the censoring probabilities (comparing between the scenarios) and the number of time points (comparing *t* = 3 and *t* = 5). When the censoring probability is high but the number of time points is less than five, our proposed estimator might be more efficient than the IPCW Kaplan–Meier estimator.

### Data applications

Our proposed estimator was applied to the MEGA study data to estimate treatment group-specific risks at 5 years after randomization. As baseline covariates, we included age (years), gender, body mass index, history of hypertension and diabetes, hypercholesterolemia medication history, current smoking, current alcohol drinking, triglyceride, high-density lipoprotein cholesterol, and low-density lipoprotein cholesterol. As the time-varying covariate, we used recent TC.

After transforming our data into one record per person-time, we estimated the survival curve using our proposed estimator. First, we fitted the models for censoring *C*_*t*_, event *Y*_*t*_, and covariate *L*_*t*_. We fitted pooled logistic models for *C*_*t*_ and *Y*_*t*_, where the time-varying intercept was included as a restricted cubic spline with 4 knots at 1–4 years after randomization. We fitted a linear model for *L*_*t*_. By fitting the pooled logistic model for *Y*_*t*_, classical risk factors for CHD (age, male, hypertension, and diabetes) were found to be the prognostic factors (Additional file 1, Appendix D). By fitting the model for *C*_*t*_, those without hypertension, diabetes, or no history of medication for hyperlipidemia, tended to be censored before the end of the follow-up period (Additional file 1, Appendix E). Unexpectedly, time-varying TC hardly affected the event or censoring after adjusted for those important baseline covariates; therefore, baseline-conditional independence assumption rather than conditional independence assumption might be plausible in the MEGA study.

We estimated the risk of CHD incidence at 5 years from randomization using the Kaplan–Meier estimator, IPCW Kaplan–Meier estimator, the parametric g-formula estimator, and our proposed estimator. The results are shown in Table 3. In the MEGA study dataset, the risk of CHD estimated using the usual Kaplan–Meier estimator and the risk estimated by other estimators were very similar. This may be due to the small impact of dependent censoring in the MEGA study and correctness of model specification for censoring and events. Because the ordinal Kaplan–Meier estimator showed similar results as the other three estimators that adjust for the possible dependent censoring, the impact of dependent censoring must be very mild. If the censoring model or event model was misspecified, the results from the other three estimators might be more different. Therefore, the results from the three estimators may indicate that the postulated models were nearly correctly specified. The estimated confidence interval of the parametric g-formula estimator was narrower than the other estimators. The estimated confidence interval for the risk of diet + pravastatin group of our proposed estimator was narrower than the IPCW Kaplan–Meier estimator.

**Table 3:** Risk of coronary heart diseases in the MEGA study at 5 years after randomization

	Diet group		Diet + pravastatin group			
Method	Risk (%)	95% CI	Risk (%)	95% CI	Risk Ratio	95% CI
Kaplan–Meier^a^	2.34	(1.90, 2.89)	1.63	(1.26, 2.11)		
IPCW Kaplan–Meier	2.39	(1.91, 2.95)	1.60	(1.19, 2.10)	0.68	(0.40, 1.06)
Parametric g-formula	2.36	(1.97, 3.01)	1.66	(1.30, 2.05)	0.71	(0.47, 0.98)
Proposed estimator	2.38	(1.91, 2.95)	1.61	(1.22, 2.06)	0.69	(0.42, 1.03)

*CI* confidence interval

^a^The confidence intervals of the Kaplan–Meier estimator was obtained using the Greenwood formula

## Discussion

In this paper, we proposed a doubly robust estimator of risk that adjusts for dependent censoring due to time-varying covariates in RCT settings. The novelty of our proposed estimator is as an extension of the existing estimator [9, 19] for more complex data with *t* > 1 and time-varying covariates. The IPCW Kaplan–Meier estimator is routinely used in the analysis of RCTs for the purpose of adjusting for dependent censoring with time-varying covariates measured throughout the follow-up period. We have also provided SAS codes (Additional file 1, Appendix B) and an example of simulation data (Additional file 2). The important property of our proposed estimator is the double protection against model misspecification. Because risk factors for the endpoints are often identified before the beginning of the RCT, by measuring them longitudinally at as many time points as possible and by using them when constructing the models, we are in a better position to approximate the true regression function.

The second property of our proposed estimator is the efficiency recovery over the IPCW Kaplan–Meier estimator, as shown in the simulation study. The degree of efficiency recovery could depend on either the censoring probability, event probability, the dependency of variables, or all of these factors combined. As studied previously [8], we considered that the censoring probability is an important factor. Further studies will involve understanding the factors that affect the degree of efficiency recovery using further simulations. In the simulation study and analysis of the MEGA study, the parametric g-formula estimator outperformed regarding efficiency. This phenomenon was expected because the asymptotic variance of the classical doubly robust estimator is no smaller than that of the g-formula estimator [26].

Our estimator relies on the assumption that censoring and event time are independent conditional on observed covariates including time-varying ones. However, in a situation that censoring and event time are not independent even if we condition on time-varying covariates, our proposed estimator and other existing estimators cannot correct for selection bias. We also need the assumption of correct model specification. We need to incorporate the covariates that affect both event and censoring probabilities, and moreover, we need to specify the model form that approximates the true regression function.

In this study, we considered the estimation of a survival function in a specific group. If we compare two or more survival functions that may be observed with different interventions, we also need an additional exchangeability assumption (or the no-unmeasured confounders assumption) between the intervention groups [27]. In the simulations and data analysis, the exchangeability assumption is satisfied at baseline owing to the randomized design. In a future study, it will be interesting to extend our estimator into the observational study setting [24, 25].

All the estimators in this study can be applied to right-censored data. We consider that our proposed estimator cannot be applied to the data with interval or left-censored data in its current form. With those censoring, we know that an event has occurred only before a specific time. In this situation, how to predict event probability and how to weight uncensored subjects are not obvious. Note that the MEGA study corrects exact event time, so we consider that interval censoring or left censoring is absent in the real data.

There are several reasons for censoring in the MEGA study, as shown in Table 1. We treated refusal of follow-up, death by causes other than CHD, and loss to follow-up as reasons for censoring in the censoring model. Three estimators, including our proposed estimator, assessed the hypothetical survival function when there was no censoring. It may be meaningful to consider whether a survival function can be obtained if refusal to follow-up and loss to follow-up did not occur. When we separately accounted for the two reasons for dropouts, the survival curve was similar to the one using the Kaplan–Meier method [28]. However, death by causes other than CHD needs additional consideration, because it is difficult to cease such competing risks for CHD without lowering the risk of CHD. Therefore, if there was no death by causes other than CHD, the survival function would be slightly lower than we estimated. Because in the MEGA study the proportion of censoring due to death by causes other than CHD was less than 1.5%, we believe the estimated survival functions are close to the true survival function, which would be obtained if these censorings had not occurred.

There are two limitations in this study. First, we were not able to verify the assumptions with the measured data. The positivity assumption will be satisfied unless the conditional probabilities of censoring are zero for all patients at *t* = 1,…, *T.* In the analysis of the MEGA study data, there were no patients who had an estimated probability of censoring near 1 (data not shown); therefore, we considered that the positivity assumption is acceptable. Conditional independence assumption implies that the treatment group, measured baseline, and time-varying covariates can completely explain censoring. However, given a rich collection of measured prognostic factors, the conditional independence assumption can be approximated. Several clinically important prognostic factors were measured in the MEGA study, and we used all of the baseline covariates and a time-varying covariate, TC. We considered time-varying TC was important for event and censoring probability, but the hazard ratio was close to 1; therefore, the impact of dependent censoring was very mild. In the future, we need to apply our estimator to data with censoring dependent on time-varying factors. The second limitation was the range of the simulation study. Because we were interested in the statistical properties of the estimators with fitted correct/incorrect models, the behavior of the estimators when other assumptions, such as positivity, were violated is unknown. We need further simulation studies to understand the performance of the estimators.

## Conclusions

The proposed estimator is useful for the estimation of risk if censoring affected by time-varying risk factors occurred because of the doubly robust property and statistical efficiency over the IPCW Kaplan–Meier method.

## Supplementary information

**Additional file 1.**

**Additional file 2.**

## Data Availability

The SAS code is available in Additional file 1 (Appendix B), and an example of simulated dataset is available in Additional file 2. Real data were originally published in Nakamura et al. [5].

## Abbreviations

CHD: Coronary heart disease
IPCW: Inverse probability-of-censoring weighted
MEGA: Management of Elevated Cholesterol in the Primary Prevention Group of Adult Japanese
RCT: Randomized controlled trial;
TC: Total cholesterol
