# Supplementary material for: Doubly robust estimator of risk in the presence of censoring dependent on time-varying covariates: application to a primary prevention trial for coronary events with pravastatin
Source: BMC Med Res Methodol. 2020 Jul 31;20:204. doi: 10.1186/s12874-020-01087-8 (PMC7395418; doi:10.1186/s12874-020-01087-8)
Supplement: Supplementary file 1 — Additional file 1. [file 12874_2020_1087_MOESM1_ESM.pdf]

Appendix A. Double robustness

The proposed estimator has the double robustness; consistent if either the model for the IPCW Kaplan-Meier estimator or the models for the parametric g-formula estimator, but not necessarily both, are correctly specified. This is demonstrated below. Our proposed estimator first needs to fit censoring, event, and covariate models like equations (4) (5) and (6). Here, the maximum likelihood estimation for logistic models and linear models is used. For example, the likelihood for model (4) is $\prod_{i=1}^{n}\left[\prod_{k:\,timing\ of\ censoring}\exp(\alpha_{0k}+\alpha_1 V_i+\alpha_2 L_{i.k-1})^{-1}\prod_{j:at\ Risk}\left(1+\exp\left\{-\left(\alpha_{0j}+\alpha_1 V_i+\alpha_2 L_{i,j-1}\right)\right\}\right)\right]$. By virtue of maximum likelihood estimates, $(\hat{\alpha},\hat{\beta},\hat{\gamma})$converges in probability to $(\alpha^*,\beta^*,\gamma^*)$, each probability limit equals the true parameter value when the corresponding parametric model is correctly specified. Subsequently, the estimated discrete event hazard and uncensored probability converge in probability to $\Pr\left(Y_t=1\middle|\bar{Y}_{t-1}=0,V,\bar{L}_{t-1};\beta^*,\gamma^*\right)$ and $\pi_t(\alpha^*)$, respectively. As $n\to\infty$, (7) converges in probability to

$$E\left[\frac{(1-C_t)Y_t}{\pi_t(\alpha^*)}-\frac{(1-C_t)-\pi_t(\alpha^*)}{\pi_t(\alpha^*)}\Pr\left(Y_t=1\middle|\bar{Y}_{t-1}=0,V,\bar{L}_{t-1};\beta^*,\gamma^*\right)\middle|\bar{Y}_{t-1}=0\right]$$

$$=E\left(Y_t\middle|\bar{Y}_{t-1}=0\right)$$

$$+E\left[\frac{(1-C_t)-\pi_t(\alpha^*)}{\pi_t(\alpha^*)}\left\{Y_t-\Pr\left(Y_t=1\middle|\bar{Y}_{t-1}=0,V,\bar{L}_{t-1};\beta^*,\gamma^*\right)\right\}\middle|\bar{Y}_{t-1}=0\right]$$

$$=E\left(Y_t\middle|\bar{Y}_{t-1}=0\right)$$

$$+E\left[\frac{(1-C_t)-\pi_t(\alpha^*)}{\pi_t(\alpha^*)}\middle|\bar{Y}_{t-1}=0,V,\bar{L}_{t-1}\right]$$

$$\times E\left[Y_t-\Pr\left(Y_t=1\middle|\bar{Y}_{t-1}=0,V,\bar{L}_{t-1};\beta^*,\gamma^*\right)\middle|\bar{Y}_{t-1}=0,V,\bar{L}_{t-1}\right]$$

where the second equality follows from the assumption (2). Therefore, the estimator is consistent for the discrete event hazard at t, if either censoring model is correct so that $\pi_t(\alpha^*)$ equals $E\left[1 - C_t \middle| \bar{Y}_{t-1} = 0, V, \bar{L}_{t-1}\right]$, or event and covariate models are correct so that $\Pr\left(Y_t = 1 \middle| \bar{Y}_{t-1} = 0, V\ , \bar{L}_{t-1}; \beta^*, \gamma^*\right)$ equals $E\left[Y_t \middle| \bar{Y}_{t-1} = 0, V, \bar{L}_{t-1}\right]$.

Appendix B. SAS code of the proposed estimator

Note: An example dataset *observed1* is provided as Supplementary file 2.

```
/****

SAS code of proposed estimator (Kawahara T, et al.)


NOTES: This code can be applied for data formatted like observed1 dataset
observed1 dataset is formatted as 1 record per 1 subject
observed1 contains following variables :
  id : subject id
  b0,  b1, v, l0: baseline covariates
  r : randomized treatment group (0 or 1) (current sort key)
  l1, l2, l3, l4: time-dependent covariate (t= 1, 2, 3, 4)
  ut (t=0, 1, 2, 3, 4) = 1 if lt < 0, ut = 0 otherwise
  delta : censoring indicator (takes 1 if the subject was censored, 0 if the
subject had event)
  t : observed time (time-to-event or censoring)

****/

/*Create 1 record per person-time*/
data persontime1;
        set observed1;
        month_t=ceil(t*12);
        do month=1 to month_t;
                year=ceil(month/12)-1;
                censor=0;
                event=0;
                if month=month_t and delta=1 then do;
                        censor=1;
                        event=0;
```

```
                year=ceil(month/12)-1;
            end;
            else if month=month_t and delta=0 then do;
                censor=.;
                event=1;
            end;
        output;
        end;
run;
data persontime2;
        set persontime1;
        if year = 0 then do; l = l0; u = u0; end;
        if year = 1 then do; l = l1; u = u1; end;
        if year = 2 then do; l = l2; u = u2; end;
        if year = 3 then do; l = l3; u = u3; end;
        if year = 4 then do; l = l4; u = u4; end;
run;

data _null_;
        call symput('ol', 'v u r');
        call symput('y_lin1', 'beta01+beta02*v+beta03*estu0+beta04*r');
        call symput('y_lin2', 'beta01+beta02*v+beta03*estu1+beta04*r');
        call symput('y_lin3', 'beta01+beta02*v+beta03*estu2+beta04*r');
        call symput('y_lin4', 'beta01+beta02*v+beta03*estu3+beta04*r');
        call symput('y_lin5', 'beta01+beta02*v+beta03*estu4+beta04*r');
        call symput('outdrop', 'beta01--beta04');
        call symput('cl', 'v u r');
        call symput('drc_lin1', 'beta11+beta12*v+beta13*estu0+beta14*r');
        call symput('drc_lin2', 'beta11+beta12*v+beta13*estu1+beta14*r');
        call symput('drc_lin3', 'beta11+beta12*v+beta13*estu2+beta14*r');
        call symput('drc_lin4', 'beta11+beta12*v+beta13*estu3+beta14*r');
        call symput('cendrop', 'beta11--beta14');
        call symput('covl', 'year r*year b0 b1*year');
        call
symput('cov_lin1','beta11+beta12*1+beta13*r*1+beta14*b0+beta15*b1*1');
        call
symput('cov_lin2','beta11+beta12*2+beta13*r*2+beta14*b0+beta15*b1*2');
        call
```

```
symput('cov_lin3','beta11+beta12*3+beta13*r*3+beta14*b0+beta15*b1*3');
	call
symput('cov_lin4','beta11+beta12*4+beta13*r*4+beta14*b0+beta15*b1*4');
	call symput('covdrop', '_NAME_--beta15');

run;



/*EVENT model*/
proc logistic data=persontime2;
	model event(event='1') = &ol.  ; /* here explanatory variables for event
models are specified*/
	ods output ParameterEstimates=param_y_0;
run;
proc transpose data=param_y_0(keep=estimate) out=param_y(drop=_NAME_ _LABEL_)
prefix=beta0;run;

/**COVARIATE model*/
proc glm data=persontime2;
	model l = &covl. ; /* here explanatory variables for covariate models are
specified*/
	ods output parameterestimates=param_l_10;
run;quit;
proc transpose data=param_l_10(keep=estimate) out=param_l prefix=beta1;run;

/*CENSORING model*/
proc logistic data=persontime2;
	model censor(event='1')= &cl. ; /* here explanatory variables for
censoring models are specified*/
	ods output parameterestimates=param_c_1;
	where month in (12 24 36 48);
run;
proc transpose data=param_c_1(keep=estimate) out=param_c(drop=_NAME_ _LABEL_)
prefix=beta1;run;



data dr01;
	set observed1;
```

```
        year=1; output;
        year=2; output;
        year=3; output;
        year=4; output;
        year=5; output;
run;
data dr02;
        call streaminit(1);
        if _n_=1 then set param_l;
        set dr01;
        estl0=l0;
        estl1=&cov_lin1.+rand('normal',0,1);
        estl2=&cov_lin2.+rand('normal',0,1);
        estl3=&cov_lin3.+rand('normal',0,1);
        estl4=&cov_lin4.+rand('normal',0,1);
        estu0=u0;
        if u1 ne . then estu1=u1; else estu1=(estl1<0);
        if u2 ne . then estu2=u2; else estu2=(estl2<0);
        if u3 ne . then estu3=u3; else estu3=(estl3<0);
        if u4 ne . then estu4=u4; else estu4=(estl4<0);
        estsql0=sql0;
        estsql1=exp(estl1);
        estsql2=exp(estl2);
        estsql3=exp(estl3);
        estsql4=exp(estl4);
        drop _NAME_ _LABEL_ &covdrop.;
run;
data dr03;
        if _n_=1 then set param_y;
        set dr02;
        ests1=1/(1+exp(&y_lin1.))**12;
        ests2=1/(1+exp(&y_lin2.))**12;
        ests3=1/(1+exp(&y_lin3.))**12;
        ests4=1/(1+exp(&y_lin4.))**12;
        ests5=1/(1+exp(&y_lin5.))**12;
        surv1=ests1;
        surv2=ests2;
        surv3=ests3;
```

```
        surv4=ests4;
        surv5=ests5;
        if year = 1 then risk=1-surv1;
        if year = 2 then risk=1-surv2;
        if year = 3 then risk=1-surv3;
        if year = 4 then risk=1-surv4;
        if year = 5 then risk=1-surv5;
        drop &outdrop.;
run;
data dr04;
        if _n_=1 then set param_c;
        set dr03;
        obsy = 1 - delta ;
        if t>=year and obsy=0 then atrisk=1;
        else if ceil(t)>=year and obsy=1 then atrisk=1;
        else if ceil(t)<year and obsy=1 then atrisk=0;
        else if t<year and obsy=0 then atrisk=0;
        if year=1 then estobs=1;
        else if year=2 then estobs=(1/(1+exp(&drc_lin1.)));
        else if year=3 then estobs=(1/(1+exp(&drc_lin1.))*1/(1+exp(&drc_lin2.)));
        else if year=4 then
estobs=(1/(1+exp(&drc_lin1.))*1/(1+exp(&drc_lin2.))*1/(1+exp(&drc_lin3.)));
        else if year=5 then
estobs=(1/(1+exp(&drc_lin1.))*1/(1+exp(&drc_lin2.))*1/(1+exp(&drc_lin3.))*1/(1+ex
p(&drc_lin4.)));
        if obsy=1 and ceil(t)=year then event=1;else event=0;

        if atrisk=1 then dr=event/estobs-(1-estobs)*risk/estobs;
               else if atrisk=0 then dr=risk;
        weight=1/estobs;

        drop &cendrop.;
        if ceil(t)<year and obsy=1 then z=0; else z=1;
run;
proc means data=dr04;
        where z=1;
        var dr;
        class r year;
```

```
        output out=dr05(where=(r ne . and year ne .)) mean=/autoname;
run;
data dr06;
        set dr05;
        by r year;
        if first.r then survivalprob=1;
        retain survivalprob;
        survivalprob=(1-dr_mean)*survivalprob;
        risk=1-survivalprob;
        keep r year survivalprob risk;
run;
title "Proposed estimator";
  proc print data=dr06;run;
```

Appendix C. Dependence between the event and the censoring time in simulation study.

To assess the direct dependence between the event and the censoring times, we generated n = 2,000,000 datasets and obtained potential survival time distribution between censored and uncensored subjects in scenario 1. We below show their distribution among $R = 0$.

		Potential survival time since the time point				
		**P5**	**P25**	**P50 (Median)**	**P75**	**P95**
***t* = 1**						
	Uncensored	1.33	8.38	24.06	62.62	197.85
	Censored	0.85	5.12	14.05	33.06	111.96
***t* = 2**						
	Uncensored	1.20	7.61	22.15	58.37	190.34
	Censored	0.70	4.31	11.80	29.28	101.07
***t* = 3**						
	Uncensored	1.12	7.11	21.02	55.38	184.79
	Censored	0.64	3.77	10.36	26.31	90.58
***t* = 4**						
	Uncensored	1.08	6.81	20.09	53.28	180.14
	Censored	0.59	3.48	9.40	24.02	84.47

In the table, P5 is 5-percentile of the survival distribution, and the others similarly meant percentiles.

As shown from these tables, censored subjects have shorter potential survival than uncensored subjects.

Appendix D. Parameter estimates of fitted pooled logistic model for CHD event

	Diet		Diet + pravastatin	
	HR	95%CI	HR	95%CI
Age (years)	1.06	1.02, 1.09	1.04	1.00, 1.08
Sex (women)	0.38	0.22, 0.68	0.40	0.19, 0.82
BMI (kg/m^2)	0.92	0.85, 1.00	1.01	0.93, 1.09
Hypertension	1.94	1.21, 3.10	2.21	1.21, 4.04
Diabetes	2.37	1.50, 3.72	2.61	1.49, 4.58
Hypercholesterolemia medication history	0.82	0.45, 1.51	1.93	0.95, 3.96
Current smoker	1.23	0.69, 2.17	1.02	0.51, 2.07
Current drinker	0.89	0.51, 1.57	0.91	0.45, 1.83
TG (mg/dl)	1.00	1.00, 1.01	1.00	1.00, 1.01
HDL-C (mg/dl)	1.01	0.99, 1.03	1.00	0.97, 1.02
LDL-C (mg/dl)	1.00	0.98, 1.02	0.98	0.95, 1.02
Time-Dependent TC (mg/dl)	1.00	0.99, 1.01	1.00	0.98, 1.01

BMI, body mass index; TC, total cholesterol; TG, triglyceride; HDL-C, high density lipoprotein cholesterol; LDL-C, low density lipoprotein cholesterol; HR, hazard ratio; CI, confidence interval

Appendix E. Parameter estimates of fitted pooled logistic model for censoring

	Diet		Diet + pravastatin	
	HR	95%CI	HR	95%CI
Age (years)	1.00	0.98, 1.01	1.02	1.00, 1.03
Sex (women)	1.07	0.79, 1.44	0.93	0.72, 1.19
BMI (kg/m^2)	0.98	0.95, 1.02	1.01	0.98, 1.04
Hypertension	1.06	0.86, 1.32	0.85	0.69, 1.06
Diabetes	0.79	0.62, 1.02	0.66	0.51, 0.86
Hypercholesterolemia medication history	0.76	0.56, 1.02	0.79	0.57, 1.10
Current smoker	1.01	0.72, 1.40	0.83	0.62, 1.09
Current drinker	0.96	0.74, 1.27	1.16	0.91, 1.47
TG (mg/dl)	1.00	1.00, 1.00	1.00	1.00, 1.00
HDL-C (mg/dl)	1.00	0.99, 1.01	1.00	0.99, 1.01
LDL-C (mg/dl)	1.00	0.99, 1.01	1.00	0.99, 1.01
Time-Dependent TC (mg/dl)	1.00	0.99, 1.00	1.00	1.00, 1.01

BMI, body mass index; TC, total cholesterol; TG, triglyceride; HDL-C, high density lipoprotein cholesterol; LDL-C, low density lipoprotein cholesterol; HR, hazard ratio; CI, confidence interval
